# The Microbial Perspective: A Systematic Literature Review on Hypertension and Gut Microbiota

**DOI:** 10.3390/nu16213698

**Published:** 2024-10-30

**Authors:** Alexandros Tsiavos, Christina Antza, Christina Trakatelli, Vasilios Kotsis

**Affiliations:** 3rd Department of Internal Medicine, Aristotle University, Hypertension-24 h Ambulatory Blood Pressure Monitoring Center, Papageorgiou Hospital, 56429 Thessaloniki, Greece; alextsiavos13@gmail.com (A.T.); kris-antza@hotmail.com (C.A.); cmtrakatelli@gmail.com (C.T.)

**Keywords:** gut microbiota, hypertension, meta-analysis, systematic literature review, healthy controls

## Abstract

Background: Understanding the causes of hypertension is important in order to prevent the disease. Gut microbiota (GM) seems to play an important role, but the detailed physiology remains elusive, with alpha diversity being the most studied indicator. Objectives: This review aimed to systematically synthesize data on gut microbiota (alpha diversity) and hypertension. Methods: Databases, including MEDLINE/PubMed, Scopus, and EMBASE, and citations were systematically queried. We retrieved articles reporting the association between gut microbiota and hypertension. A valid critical appraisal tool was also used to investigate the quality of the included studies. Results: Eighteen eligible studies met our inclusion criteria. In this report, we focused on the following indices of alpha diversity: Shannon, Chao1, Simpson, and Abundance-based Coverage Estimator (ACE) indices. Several studies observed a significantly lower Shannon index in hypertensive patients compared to the healthy control group. Nevertheless, no statistically significant difference was found for the Chao1, Simpson, and ACE indices between hypertensive patients and controls. A higher Firmicutes-to-Bacteroidetes ratio (F/B ratio) was consistently observed in hypertensive patients compared to healthy controls, indicating potential dysbiosis in the gut microbiota. Conclusions: Our systematic review indicates that hypertensive patients may exhibit an imbalance in gut microbiota, evidenced by decreased alpha diversity and an elevated F/B ratio. However, the absence of statistically significant differences in secondary diversity indices (Chao1, Simpson, and ACE) highlights the need for further research. Well-designed, large-scale studies are necessary to clarify these associations and explore the role of gut microbiota in hypertension development.

## 1. Introduction

Hypertension (HTN) constitutes a significant global public health challenge, affecting approximately one-third of the population annually [1]. Its prevalence exhibits considerable variability across different regions, highlighting notable disparities; for instance, indigenous populations such as the Yanomami in the Amazon rainforest and the Kuna in the San Blas Islands of Panama display lower rates of hypertension, whereas urbanized communities, particularly in the United States, experience elevated prevalence rates attributable to lifestyle and dietary factors [2]. Several studies have indicated a potential causal link between hypertension and various conditions, including vascular complications and ischemic heart disease [3,4].

Given the complexity of hypertension pathogenesis, elucidating its etiology is paramount for disease prevention [5]. Environmental factors and lifestyle choices are well-established determinants of hypertension, with emerging evidence suggesting a potential role of gut microbiota—microorganisms inhabiting the gastrointestinal tract—as a contributing risk factor for hypertension development [6]. Gut microbiota have been implicated in various disease states, and intriguingly, trigger biological pathways involved in the dysregulation of arterial blood pressure (BP) homeostasis in human body [6,7]. However, big data population studies are not enough to provide a clear direction in this pathophysiological correlation.

Alpha diversity is regarded as a primary metric due to its capacity to quantify both species richness and evenness within microbial communities [8]. This parameter is essential for evaluating the ecological stability and functionality of the gut microbiota, which play a pivotal role in maintaining metabolic equilibrium, immunological regulation, and the integrity of the intestinal barrier [9]. Lower microbial diversity is associated with various pathological conditions, including hypertension, making this measure a crucial tool in understanding the microbiome’s contribution to disease pathogenesis and overall health [10,11]. Consequently, it provides a holistic insight into microbiota health.

Regarding hypertension, data show differences in the gut microbiota of hypertensive patients compared to healthy controls. A recent meta-analysis by Cai et al., 2023 [12], examined the relationship between gut microbiota and hypertension. The results demonstrated a lower Shannon index among hypertensive adults compared to controls, with no significant differences observed in the ACE, Simpson, or Chao1 indices. Additionally, the F/B ratio was higher in hypertensive cases compared to controls.

In this manuscript, we systematically reviewed the literature to identify the effect of gut microbiota on blood pressure. The secondary aim was to identify whether published data are enough to prove that gut microbiota could guide further management/treatment targets of the hypertensive population or more focused research is needed.

## 2. Methods

This systematic review was conducted in accordance with the Preferred Reporting Items for Systematic reviews and Meta-Analyses (PRISMA) guidelines [13]. The protocol has been registered on the Open Science Framework (OSF), DOI 10.17605/OSF.IO/KXVGD.

### 2.1. Search Strategy

**Figure 1:**
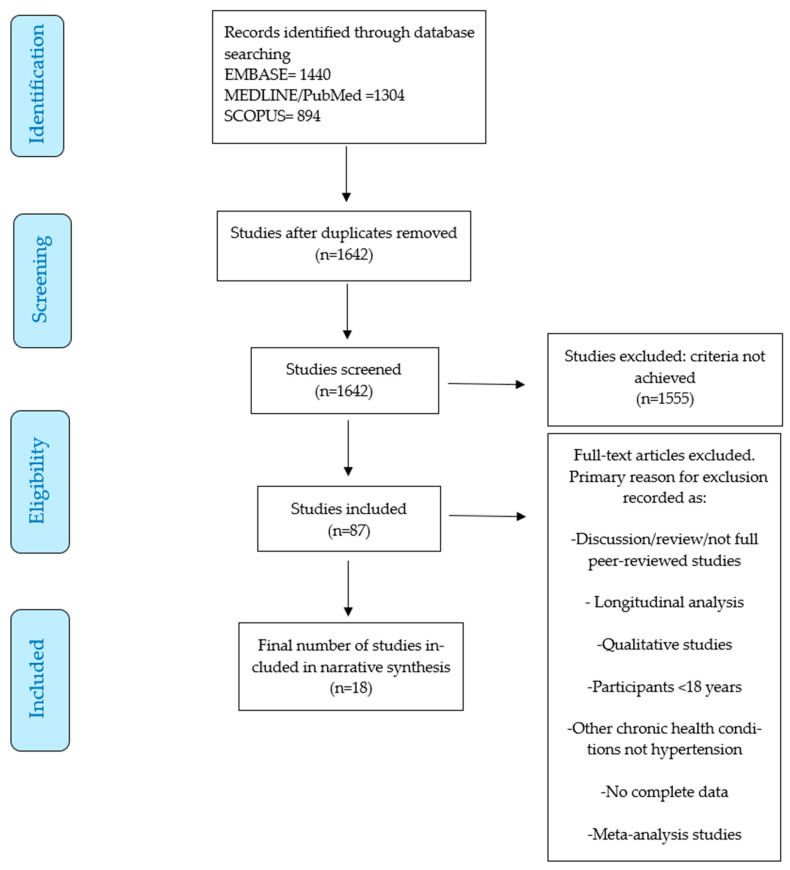
Flowchart diagram of results.

For the present study, a comprehensive literature search strategy was employed using MEDLINE (Figure 1) and other databases, including PubMed, Scopus, and EMBASE. The keywords utilized were “hypertension”, “high blood pressure”, “gut microbiota”, and “gut microflora”.

AT conducted all bibliographic searches. After the exclusion of duplicates, AT screened the titles. If the eligibility of a relevant article could not be identified initially from the title, we extracted the full text. Another investigator (CT) screened a random sample of the articles using the Rayyan software [14] (http://rayyan.qcri.org, accessed on 10 March 2023) in order to confirm consistency in the study selection process.

Any possible disagreements were resolved by a senior author, an expert in the field (CA). All systematic literature searches were performed from study inception to 31 May 2024.

### 2.2. Eligibility Criteria

#### Inclusion Criteria

We included appropriate studies based on the following criteria: (1) original research (case–control, cross-sectional, or cohort), reporting the association between hypertension and gut microbiota; (2) age ≥ 18 years old; (3) healthy controls (HCs) as control group; (4) outcome measures included gut microbiota alpha diversity indices, such as the Shannon, Chao1, ACE, and Simpson, as well as the F/B ratio; (5) English language.

### 2.3. Data Extraction

AT extracted and cross-checked the data independently while assessing the quality of studies selected for further review. CT acted as the second independent reviewer. The extracted data included the following information: citation, study location, year of publication, participant’s characteristics in the studies, and outcome.

Two researchers (AT, CT) utilized the Critical Appraisal Programme (CASP) quality assessment tool (Supplementary Material: Overview of the CASP Quality Assessment Tool) [15] in order to evaluate the quality of the included articles (Critical Appraisal Skills Programme, 2022). A third reviewer (CA) assisted in resolving discrepancies between the two researchers when necessary.

## 3. Results

A total of 1642 records were identified from the literature research across databases including Medline/PubMed, EMBASE, Scopus, and Google Scholar. Upon a detailed examination of the abstracts and titles, 87 studies were assessed for eligibility. In total, 18 articles were included in the final narrative synthesis. The process is illustrated in a flowchart diagram (Figure 1).

### 3.1. Characteristics of the Included Studies

Table 1 delineates the characteristics of the studies. All pertinent studies were published between 2017 and 2022, with preponderance (13 out of 18) emerging between 2020 and 2022. In terms of geographic distribution, the majority (10 out of 18) emanated from China, a region marked by a pronounced burden of hypertension, while the remaining 8 studies derived from the USA [3], Britain [7], Spain [16], Japan [17], Australia [5], Brazil [18], Finland [19], and the Netherlands [20]. Regarding microbiota evaluation methods, the studies primarily employed two sequencing technologies: metagenomic shotgun sequencing and 16S ribosomal RNA (16S rRNA) gene sequencing, with the latter being the most predominantly applied. Most of the studies employed a case (hypertensive patients)-control (healthy) design, while several others utilized a cohort study design, and one study adopted a cross-sectional approach.

Finally, the majority of the studies reported adjustments for age, gender, and Body Mass Index (BMI). However, there were many differences regarding other parameters such as cholesterol levels or smoking. Regarding the exclusion criteria of the studies, there were studies without even one report at all. The rest of the studies, reporting exclusion criteria, also presented differences, such as in the duration of the recent probiotic use varying from no report to 3 months. Adjustment and exclusion criteria are presented in Supplementary Table S1. However, there were differentiations in many parameters, with the most important being the measurement of blood pressure as well as the guidelines followed for the cut-off values (Supplementary Table S2).

### 3.2. Quality Assessment

The studies included in our review were rigorously assessed using the CASP checklist (Supplementary Table S3) [15], which evaluates the validity of the study outcomes, their relevance to the research question, and their broader applicability in the field. Study quality was determined based on the proportion of “Yes” responses on the CASP checklist, with a threshold of two out of three positive responses required to classify a study as high-quality [15]. In our review, three studies [3,19,28] met this threshold, receiving at least two out of three positive assessments, and were consequently characterized as good quality. The remaining studies, which fell below this threshold, did not meet the criteria for high-quality classification. By adhering to this standardized assessment framework, we ensured a consistent and objective evaluation of the methodological rigor of the included studies.

### 3.3. Alteration in Gut Microbiota Diversity in Hypertension

Alpha diversity represents the species diversity within a specific sample. In this study, we evaluated four indices of alpha diversity: the Shannon index, Chao1 index, ACE index, and Simpson index.

#### 3.3.1. Shannon Index

The Shannon index, a pivotal metric for assessing alpha diversity, has been the focus of numerous studies investigating its relationship with hypertension, yielding diverse findings. An overview of these results is provided in Table 2. Li et al., 2017 [4], observed a statistically significant lower Shannon index in hypertensive patients compared to controls. This study accounted for confounding variables such as demographic factors, BMI, and lipid metabolism, while exclusions encompassed major cardiovascular diseases, metabolic disorders, and recent antibiotic or probiotic use. In a similar vein, Yan et al., 2017 [21], also reported significantly lower Shannon diversity in hypertensive individuals compared to controls, after adjusting for a comprehensive range of factors, including age, gender, BMI, lipid profile, and fasting blood glucose (FBG). These results highlight a possible association between reduced microbial diversity and the hypertensive state.

However, several studies have reported no statistically significant differences in Shannon diversity between hypertensive and normotensive individuals. Dan et al., 2019 [23], found no significant differences in Shannon diversity between the two groups, even after controlling for variables such as gender, age, BMI, waist-to-hip ratio, metabolic profile, and lipid levels. Similarly, Takagi et al., 2020 [17], reported no substantial differences in Shannon diversity, despite adjusting for age and gender, with participants excluded for gastrointestinal diseases, malignancies, metabolic disorders, and probiotic use.

In a study conducted in Brazil, Silveira-Nunes et al., 2020 [18], did not identify any significant discrepancies in Shannon diversity between hypertensive and normotensive participants, even after accounting for age and gender, while exclusions parameters encompassed infections, autoimmune diseases, and recent antibiotic/probiotic use. Zhu et al., 2020 [24], also reported no notable distinction in Shannon diversity between hypertensive and control groups, despite several adjustments for demographic variables, metabolic profile, and medical history. Calderón-Pérez et al., 2020 [16], reached similar conclusions, with no statistically significant differences observed in Shannon diversity after adjusting for key confounders, including gender, physical activity, body composition, and lipid levels.

Further research corroborated these findings. Wang JM et al., 2021 [26], found no prominent discrepancies in Shannon diversity, even after adjusting for a range of clinical and metabolic variables such as age, gender, BMI, and blood markers. Similarly, both Nakai et al., 2021 [5], and Wan et al., 2021 [25], reported no significant differences in Shannon diversity between hypertensive and control groups, despite adjustments for confounders such as age, gender, and BMI.

Notably, Liu Y et al., 2021 [27], observed lower Shannon diversity in hypertensive patients compared to healthy controls, although this finding did not reach statistical significance. This study accounted for confounding factors such as gender, BMI, lipid profile, smoking habits, and alcohol consumption, with exclusions for gastrointestinal diseases and recent antibiotic use. Similarly, Qu et al., 2022 [29], found no significant differences in Shannon diversity, even after adjustments for age, gender, BMI, and education status.

In a series of cohort studies, Wang Y et al., 2021 [28], reported no statistically significant association between the Shannon index and arterial hypertension. The analysis accounted for demographic, dietary, physical, and renal factors, reflecting the comprehensive approach in controlling for these confounders. Furthermore, Sun et al., 2020 [3], identified an inverse association between gut microbial diversity, measured using the Shannon index, and arterial hypertension. However, this association did not reach statistical significance after adjusting for BMI, suggesting that obesity may influence the relationship between microbial diversity and hypertension. Additional variables, such as demographics, lifestyle factors, and medication use, were also adjusted for, providing a more comprehensive control of confounding factors. Subsequently, Verhaar et al., 2020 [20], observed an association between the composition of gut microbiota and blood pressure, although the Shannon index did not show statistically significant differences between participants with arterial hypertension and those without. The analysis was adjusted for gender, age, BMI, renal function, and medication use, while exclusions involved recent antibiotic use and gastrointestinal issues. Moreover, Palmu et al., 2020 [19], noted an inverse association between the Shannon index and both systolic (SBP) and diastolic blood pressure (DBP) in models adjusted for age and sex. However, this association was not statistically significant after accounting for additional factors such as BMI, smoking habits, and cardiovascular risk factors, indicating that these variables may have influenced the observed association. Finally, Jackson et al., 2018 [7], found no statistically significant differences in the Shannon index between participants with and without arterial hypertension. The analysis was adjusted for age, BMI, and technical variables related to DNA sequencing, but no significant association was identified, suggesting that microbial diversity, as measured by the Shannon index, may not be strongly affected by hypertension.

#### 3.3.2. Chao1 Index

The studies investigating the Chao1 index, as summarized in Table 3, provide consistent results regarding microbial richness in hypertensive and normotensive populations. Dan et al., 2019 [23]; Calderón-Pérez et al., 2020 [16]; Nakai et al., 2021 [5]; Wan et al., 2021 [25]; Wang JM et al., 2021 [26]; and Qu et al., 2022 [29], uniformly reported no statistically significant differences in the Chao1 index between hypertensive and normotensive groups. The absence of significant differences in microbial richness, evaluated with the Chao1 index, suggests that hypertensive status does not substantially influence overall species richness within the gut microbiota. Despite variations in study design, population characteristics, and geographic settings, the consistent findings across these investigations indicate that the Chao1 index remains stable in relation to hypertension.

#### 3.3.3. ACE Index

The studies evaluating the ACE index consistently demonstrate a lack of statistically significant differences in microbial richness between hypertensive and normotensive groups. A detailed summary of these findings is available in Table 4. Research conducted by Dan et al., 2019 [23]; Wang JM et al., 2021 [26]; and Qu et al., 2022 [29], all reported similar findings, indicating no substantial differences in microbial diversity across various populations. These studies collectively highlight that hypertensive status does not appear to significantly impact microbial richness, as measured by the ACE index, across different sample types, further corroborating the stability of microbial richness in relation to hypertensive conditions.

#### 3.3.4. Simpson Index

The studies investigating the Simpson index, as outlined in Table 5, consistently indicate no statistically significant differences in microbial diversity between hypertensive and normotensive populations. Dan et al., 2019 [23], observed no notable differences in the Simpson index between hypertensive and control groups. Similarly, Wang JM et al., 2021 [26], identified no substantial deviations in Simpson index values. Nakai et al., 2021 [5], and Wan et al., 2021 [25], also reported no discernible differences in microbial diversity between hypertensive and control cohorts. Liu Y et al., 2021 [27], noted a minor, but statistically insignificant difference in Simpson index values between hypertensive and healthy individuals. Qu et al., 2022 [29], likewise found no significant discrepancies in the Simpson index when comparing hypertensive cases to healthy controls, concluding that the observed variations were minimal and did not indicate a substantial influence of hypertension on microbial diversity.

#### 3.3.5. F/B Ratio

The studies investigating the Firmicutes/Bacteroidetes ratio, as summarized in Table 6, reveal varying outcomes regarding its association with hypertensive status. Li et al., 2017 [4], reported no significant differences in the F/B ratio between hypertensive patients and healthy controls. Similarly, Zhu et al., 2020 [24], observed no substantial discrepancies in the F/B ratio after several parameters were accounted for. Silveira-Nunes et al., 2020 [18], also identified no significant variations in the F/B ratio. In contrast, Mushtaq et al., 2019 [22], reported a higher F/B ratio among hypertensive individuals, after accounting for demographic factors, weight, lipid levels, and co-existing medical history. Likewise, Wang JM et al., 2021 [26], noted a higher F/B ratio in hypertensive patients compared to healthy controls. These findings highlight the variability across studies, with some indicating discrepancies in gut microbial balance associated with hypertension, while others observe no significant differences, reflecting the complex interplay between the gut microbiome and hypertensive pathology.

Interestingly, when stratifying the results by country of origin and methodology, it becomes apparent that Chinese studies employing 16S rRNA sequencing report a statistically significant difference in the Firmicutes/Bacteroidetes ratio in hypertensive individuals compared to controls. In contrast, the Brazilian study [18], which also utilized 16S rRNA sequencing, did not observe a significant difference in the F/B ratio. This discrepancy is likely attributable to underlying differences in dietary patterns, environmental factors, or genetic diversity across populations, rather than from the sequencing methodology itself.

## 4. Discussion

In the present paper, we conducted a comprehensive review of the literature investigating the association between gut microbiota and hypertension. To our knowledge, this is the first study to systematically identify and critically analyze studies regarding this association. Several studies showed that gut microbiota play a role in hypertension. Overall, the studies exhibited adequate quality, incorporating adjustments for certain parameters of gut microbiota; however, they did not consider other critical factors, which may significantly affect the findings. Furthermore, there were no common methodologies regarding the measurement of BP as well as guidelines followed for the BP cut-off values.

Emerging evidence suggests that alpha diversity in gut microbiota may play a significant role in the pathogenesis of hypertension. Diminished microbial diversity compromises the production of short-chain fatty acids (SCFAs), which are essential for maintaining vascular integrity and regulating inflammatory responses [30]. SCFAs, such as butyrate, help modulate immune function and maintain the gut barrier [31]. When alpha diversity decreases, gut permeability increases, allowing bacterial endotoxins to enter circulation, triggering systemic inflammation and oxidative stress [32]. These processes lead to endothelial dysfunction, impairing vasodilation and promoting vasoconstriction, both of which contribute to elevated blood pressure. Additionally, an imbalance in microbial composition, including an increased Firmicutes-to-Bacteroidetes ratio, exacerbates these effects by promoting chronic inflammation and metabolic disturbances [11,33]. Therefore, preserving microbial diversity is essential to mitigating the risk and progression of hypertension.

The microbial ecosystem is influenced by numerous factors, including geographic region, ethnicity and age. Specifically, a previous study demonstrated that black patients with hypertension exhibited a higher prevalence of treatment-resistant hypertension and simultaneously distinct gut microbiota profiles compared to white patients [34]. Moreover, another study demonstrated significant differences in gut microbiota composition across various ethnic groups, underscoring the potential impact of ethnicity on gut microbiota profiles [35]. Therefore, the study population may contribute to heterogeneity in the findings.

The HELIUS study [20] reported that gut microbiota accounted for approximately 4.5% of the systolic blood pressure variance, with notable variations across different ethnic groups. The highest explained variance was observed among Dutch participants, where gut microbiota composition accounted for 4.8% of the variance. In contrast, much lower contributions were observed among South-Asian Surinamese, African Surinamese, Ghanaian, Moroccan, and Turkish participants, where gut microbiota explained less than 0.8% of the variance. These findings suggest that the influence of gut microbiota on blood pressure regulation may vary significantly across different ethnic groups.

Apart from the study population previously mentioned, the outcomes of various published studies on gut microbiota composition may also be influenced by different lifestyle factors. The literature suggests the important role of carbo and Mediterranean diet on gut alterations, as well as the use of probiotics [36,37,38,39,40]. For example, the consumption of a high-fiber diet—a dominant characteristic of the Mediterranean diet—induces substantial alterations in gut microbiota composition, both in rodents and humans, resulting in a reduction in Firmicutes and an increase in Bacteroidetes [38]. Also, the consumption of cheese in modest amounts is recommended within the Mediterranean diet, and this seems to also have an impact on gut microbiota, as well as yogurt [39]. At this point, prebiotics have been reported to reduce the pathogens from 30% to 80% of initial challenges [40]. Furthermore, the link with alcohol-induced gut microbiota dysbiosis has been investigated, showing a possible correlation [41]. Physical activity seems to also be important. A recent meta-analysis showed that exercise changes the alpha diversity of adults, increasing the Shannon index and Firmicutes and decreasing Bacteroidetes [42]. Finally, even sleep quality plays a crucial role, with both sleep fragmentation and short sleep duration being associated with gut dysbiosis [43]. The studies included in our review, accounting for lifestyle factors such as dietary habits, physical activity, and use of probiotics, are limited [3,5,16,19,27,28]. Therefore, hypertensive patients and healthy controls may present imbalanced characteristics of these parameters, potentially contributing to the heterogeneity of the results.

However, metabolic parameters such as blood glucose levels, lipid profile, age, and BMI were frequently adjusted for, given their established association with both hypertension and gut microbiota imbalances [4,16,21,23,26].

The results of our study are in accordance with previously published data. A previous systematic review and meta-analysis of 19 studies, investigating differences in gut microbiota between hypertensive cases and controls, also detected statistically significant lower gut microbiota diversity among hypertensive patients [12]. Specifically, the study reported lower Shannon index for hypertensive cases, which is consistent with our findings and those of Yang et al. [44].

Regarding the Chao1 index, our systematic review did not provide a statistically substantial difference between hypertensive patients and controls. Similarly, the previous meta-analysis did not report a significant discrepancy in the Chao1 index between these two groups, suggesting that there is no substantial variation in the diversity of gut microbiota in hypertensive individuals [12]. Moreover, both the ACE and Simpson indices did not exhibit any significant differences, aligning with the findings of the meta-analysis [12] and indicating that microbial richness and evenness remain largely unaffected by the hypertensive status.

The major strength of this systematic literature review is the employment of a comprehensive strategy involving multiple sources. We also employed a valid quality assessment tool (CASP) for a rigorous and robust critical appraisal procedure.

However, our study had the following limitations that must be taken into consideration: we did not conduct a comprehensive review of other indicators such as Beta Diversity.

Additionally, our study did not perform a quantitative data analysis; therefore, the findings of our narrative review should be interpreted with caution.

## 5. Conclusions

In conclusion, our systematic literature review suggests that hypertensive patients may exhibit an imbalance in gut microbiota, as evidenced by the majority of the existing literature. However, not all of the current studies consider important parameters that could influence gut microbiota. Further, well-designed research is indispensable for elucidating the role of gut microbiota in the development of hypertension. Future studies should be of high quality, encompass large sample sizes, and adjust for race, nutrition, inflammation, smoking, alcohol, and metabolic factors in order to provide clear results of this possible association.

## Figures and Tables

**Table 1:** Characteristics of the included studies in the systematic review.

Study	Country	Total Patients	HTN Patients	HC Patients	Study Type	Mean Age (HTN)	Mean Age (HC)	Age Diff (*p* < 0.05)	HTN (mm Hg)	HC (mm Hg)	Investigation
Li et al., 2017 [4]	China	196	99	41	Cohort	53.6	53.7	No	SBP ≥ 140 or DBP ≥ 90	SBP ≤ 125, DBP ≤ 80	Shannon F/B Ratio
Yan et al., 2017 [21]	China	120	60	60	Case- Control	57	56	No	BP ≥ 140/90	BP ≤ 120/80	Shannon
Jackson et al., 2018 [7]	Britain	2737	2737	-	Cohort	60	Not Available	No	Diagnosed	Not Available	Shannon
Mushtaq et al., 2019 [22]	China	80	50	30	Case- Control	62.5	60.5	No	Grade 3 Hypertension	Healthy Volunteers	F/B Ratio
Dan et al., 2019 [23]	China	129	67	62	Case- Control	69.3	69.5	No	SBP ≥ 140 or DBP ≥ 90	90 ≤ SBP ≤ 140, 60 ≤ DBP ≤ 90	Shannon Chao1 ACE Simpson
Calderón- Pérez et al., 2020 [16]	Spain	61	29	32	Cross- Sectional	53.7	41.1	Yes	140 ≤ SBP ≤ 159	SBP < 120	Chao1 Shannon
Takagi et al., 2020 [17]	Japan	151	97	54	Case- Control	69	65.5	No	SBP ≥ 140 or DBP ≥ 90	Controls	Shannon
Zhu et al., 2020 [24]	China	225	121	104	Case- Control	54.6	52.4	No	Grade 3 Hypertension	Controls	F/B Ratio Shannon
Silveira-Nunes et al., 2020 [18]	Brazil	80	48	32	Case- Control	65.3	63.3	No	SBP > 140, DBP > 90	Normotensive	F/B ratio Shannon
Palmu et al., 2020 [19]	Finland	6953	6953	-	Cohort	49.2	Not Available	No	SBP ≥ 140 or DBP ≥ 90	Not Available	Shannon
Verhaar et al., 2020 [20]	Netherlands	4672	4672	-	Cohort	49.8	Not Available	No	SBP > 140 or DBP > 90	Not Available	Shannon
Sun et al., 2020 [3]	USA	529	529	-	Cohort	55.3	Not Available	No	SBP ≥ 140, DBP ≥ 90	Not Available	Shannon
Nakai et al., 2021 [5]	Australia	70	23	47	Case- Control	60.3	59.2	No	European Guidelines	Normotensive	Shannon Chao1 Simpson
Wan et al., 2021 [25]	China	600	300	300	Case- Control	69.5	69.3	No	SBP ≥ 140 or DBP ≥ 90	Normal BP	Shannon Chao1 Simpson
Wang JM et al., 2021 [26]	China	108	93	15	Case- Control	61.4	56.3	No	Stage 1 Hypertension	Healthy Participants	Shannon Chao1 Simpson ACE F/B Ratio
Liu Y et al., 2021 [27]	China	52	26	26	Case- Control	56.9	50.1	Yes	SBP ≥ 140 or DBP ≥ 90	SBP ≤ 139, DBP ≤ 89	Shannon Simpson
Wang Y et al., 2021 [28]	China	1082	1082	-	Cohort	51	Not Available	No	SBP ≥ 140, DBP ≥ 90	Not Available	Shannon
Qu et al., 2022 [29]	China	97	63	34	Cohort	59.8	59.2	No	SBP ≥ 140, DBP ≥ 90	Healthy Volunteers	Shannon Chao1 Simpson ACE

**Table 2:** Shannon index.

Author	Year	Total Patients	Number of Patients in Each Group	Mean Difference	95% CI	Statistically Significant
Li et al. [4]	2017	196	HTN: 99 HC: 41	−0.40	(−0.76, −0.03)	Lower
Yan et al. [21]	2017	120	HTN: 60 HC: 60	−0.49	(−0.85, −0.12)	Lower
Dan et al. [23]	2019	129	HTN: 67 HC: 62	0.12	(−0.23, 0.47)	No difference
Silveira-Nunes et al. [18]	2020	80	HTN: 48 HC: 32	−0.35	(−0.81, 0.10)	No difference
Zhu et al. [24]	2020	225	HTN: 121 HC: 104	−0.15	(−0.41, 0.12)	No difference
Takagi et al. [17]	2020	151	HTN: 97 HC: 54	−0.32	(−0.65, 0.02)	No difference
Calderón-Pérez et al. [16]	2020	61	HTN: 29 HC: 32	0.22	(−0.29, 0.72)	No difference
Wang JM et al. [26]	2021	108	HTN: 29 HC: 15	0.00	(−0.62, 0.62)	No difference
Nakai et al. [5]	2021	70	HTN: 23 HC: 46	0.16	(−0.34, 0.66)	No difference
Wan et al. [25]	2021	600	HTN: 300 HC: 300	−0.10	(−0.26, 0.06)	No difference
Liu Y et al. [27]	2021	52	HTN: 26 HC: 26	−0.17	(−0.71, 0.37)	No difference
Qu et al. [29]	2022	97	HTN: 63 HC: 34	0.03	(−0.39, 0.44)	No difference

**Table 3:** Chao1 index.

Author	Year	Total Patients	Number of Patients in Each Group	Mean Difference	95% CI	Statistically Significant
Dan et al. [23]	2019	129	HTN: 67 HC: 62	0.18	(−0.16, 0.53)	No difference
Calderón-Pérez et al. [16]	2020	61	HTN:29 HC: 32	−0.04	(−0.54, 0.47)	No difference
Nakai et al. [5]	2021	70	HTN: 23 HC: 46	0.13	(−0.37, 0.63)	No difference
Wan et al. [25]	2021	600	HTN: 300 HC: 300	0.08	(−0.08, 0.25)	No difference
Wang JM et al. [26]	2021	108	HTN: 29 HC: 15	0.40	(−0.23, 1.03)	No difference
Qu et al. [29]	2022	97	HTN: 63 HC: 34	0.16	(−0.25, 0.58)	No difference

**Table 4:** ACE index.

Author	Year	Total Patients	Number of Patients in Each Group	Mean Difference	95% CI	Statistically Significant
Dan et al. [23]	2019	129	HTN: 67 HC: 62	0.12	(−0.22, 0.47)	No difference
Wang JM et al. [26]	2021	108	HTN: 29 HC: 15	0.44	(−0.19, 1.07)	No difference
Qu et al. [29]	2022	97	HTN: 63 HC: 34	0.16	(−0.26, 0.58)	No difference

**Table 5:** Simpson index.

Author	Year	Total Patients	Number of Patients in Each Group	Mean Difference	95% CI	Statistically Significant
Dan et al. [23]	2019	129	HTN: 67 HC: 62	0.17	(−0.18, 0.51)	No difference
Wang JM et al. [26]	2021	108	HTN: 29 HC: 15	0.25	(−0.37, 0.88)	No difference
Nakai et al. [5]	2021	70	HTN: 23 HC: 46	0.20	(−0.30, 0.70)	No difference
Wan et al. [25]	2021	600	HTN: 300 HC: 300	−0.13	(−0.29, 0.03)	No difference
Liu Y et al. [27]	2021	52	HTN: 26 HC: 26	0.47	(−0.08, 1.02)	No difference
Qu et al. [29]	2022	97	HTN: 63 HC: 34	0.03	(−0.39, 0.44)	No difference

**Table 6:** F/B ratio.

Author	Year	Total Patients	Number of Patients in Each Group	Mean Difference	95% CI	Statistically Significant
Li et al. [4]	2017	196	HTN: 99 HC: 41	−0.24	(−0.61, 0.12)	No difference
Mushtaq et al. [22]	2019	80	HTN: 20 HC: 10	4.09	(2.74, 5.43)	Higher
Zhu et al. [24]	2020	225	HTN: 121 HC: 104	0.06	(−0.20, 0.32)	No difference
Silveira-Nunes et al. [18]	2020	80	HTN: 48 HC: 32	0.42	(−0.03, 0.87)	No difference
Wang JM et al. [26]	2021	108	HTN: 29 HC: 15	1.26	(0.58, 1.94)	Higher

## Supplementary Materials

The following supporting information can be downloaded at: https://www.mdpi.com/article/10.3390/nu16213698/s1, Overview of the CASP Quality Assessment Tool; Supplementary Table S1: Adjustments and Exclusion criteria of the included studies; Supplementary Table S2: Blood pressure measurement methods and guidelines followed by the included studies; Supplementary Table S3: Critical appraisal of the included studies.

## Abbreviations

ACE Index	Abundance-based Coverage Estimator Index
BMI	Body Mass Index
BP	Blood Pressure
DBP	Diastolic Blood Pressure
FBG	Fasting Blood Glucose
F/B ratio	Firmicutes-to-Bacteroidetes ratio
GM	Gut Microbiota
HC	Healthy Control
HTN	Hypertension
SBP	Systolic Blood Pressure
SCFA	Short-Chain Fatty Acid
16S rRNA	16S ribosomal RNA
