# Supplementary material for: The Microbial Perspective: A Systematic Literature Review on Hypertension and Gut Microbiota
Source: Nutrients. 2024 Oct 30;16(21):3698. doi: 10.3390/nu16213698 (PMC11547301; doi:10.3390/nu16213698)
Supplement: Supplementary file 1 [file nutrients-16-03698-s001.zip › Overview of the CASP Quality Assessment Tool.pdf]

Overview of the CASP Quality Assessment Tool

When critically appraising a systematic literature study, three main issues need to be taken into consideration:

First section: Validity of the Study Outcomes – Assess whether the outcomes reported in the study are credible and reliable.

Second section: Nature of the Outcomes – Evaluate the relevance of the outcomes to the research question or objective.

Third section: Utility of the Outcomes – Determine the applicability of the outcomes in addressing the research question or contributing to the field of study.

The CASP tool is designed to systematically address key issues related to the evaluation of study quality, offering a structured approach to critically appraising the validity, results, and relevance of research. The first two queries are designed as straightforward questions, with a 'yes' response leading to further, more specific queries. Answers typically include 'yes', 'no', or 'can't tell,' providing a clear framework for evaluating the quality of the study. This tool is adapted according to the specific type of study being assessed.
