# Supplementary material for: The Microbial Perspective: A Systematic Literature Review on Hypertension and Gut Microbiota
Source: Nutrients. 2024 Oct 30;16(21):3698. doi: 10.3390/nu16213698 (PMC11547301; doi:10.3390/nu16213698)
Supplement: Supplementary file 1 [file nutrients-16-03698-s001.zip › Supplementary Table S1.pdf]

Supplementary Table S1. Adjustments and Exclusion criteria of the included studies.

Study	Adjustments	Adjustments via Exclusion Criteria
Li et al., 2017 [4]	Gender, Age, BMI, TC, Triglycerides, HDL, LDL, FBG	Cancer, Heart Failure, Renal Failure, Smoking habits, Stroke, Peripheral Artery Disease, Antihypertensive Treatment, Diabetes Mellitus, Hypercholesterolemia, Recent Antibiotic/Probiotic use (8 weeks)
Yan et al., 2017 [18]	Gender, Age, BMI, FBG, HDL, LDL, TC, Triglycerides	Not specified
Jackson et al., 2018 [7]	Age, BMI, Technical confounders (DNA sequencing depth, sample handling)	Not specified
Mushtaq et al., 2019 [19]	Gender, Age, Weight, TC, HDL	Acute/Chronic inflammatory diseases, Cardiovascular disease, Chronic metabolic disease, Recent Antibiotic/Probiotic use (30 days)
Dan et al., 2019 [20]	Gender, Age, BMI, Waist-to-Hip Ratio, FBG, TC, HDL, LDL, Triglycerides, Blood Urea Nitrogen, Uric Acid, Homocysteine	Mental illness, Drug abuse, Cancer, Heart failure, Renal failure, Stroke, Peripheral artery disease, Antihypertensive therapy, Recent Antibiotic/Probiotic use (8 weeks)
Calderón-Pérez et al., 2020 [21]	Gender, HDL, Triglycerides, Physical activity, Sleep quality, TC, Fat Mass, FBG, Waist Circumference, BMI, LDL, Weight	BMI ≥ 30 kg/m², Fasting glucose > 126 mg/dL, LDL > 190 mg/dL, Triglycerides > 350 mg/dL, Smoking habits, Anemia, Intestinal disorders, Recent Antibiotic/Probiotic use (3 months), Vegetarian diet
Takagi et al., 2020 [22]	Age, Gender	Gastrointestinal diseases, Antibiotics/Corticosteroids/Immunosuppressants/Proton pump inhibitors, Malignant diseases, Severe metabolic/respiratory/neurological diseases, Pregnancy, Recent Probiotic use
Zhu et al., 2020 [23]	Age, Gender, Smoking habits, BMI, TC, Triglycerides, HDL, LDL	Gastrointestinal diseases, Antibiotics/Probiotics use (3 months), Cancer, Diabetes, Obesity, Cardiovascular diseases, Renal failure, Stroke, Peripheral artery disease, NSAID use
Silveira-Nunes et al., 2020 [24]	Age, Gender	infections, Schistosoma mansoni, Autoimmune diseases, Mood disorders, Neurodegenerative diseases, Cancer, Recent Antibiotic/Probiotic use, Anemia, Leucopoenia, Use of steroids, Alcohol use, Antidepressants use
Palmu et al., 2020 [25]	Age, Gender, BMI, Smoking habits, Exercise, Diabetes, Diuretic use, Beta-blocker use, Calcium channel blocker use, Renin-angiotensin system inhibitors use	Not specified
Verhaar et al., 2020 [26]	Gender, Age, BMI, eGFR, Antihypertensive drugs, Lipid-lowering drugs treatment, Albuminuria, Diabetes, Smoking habits	Recent antibiotic use (3 weeks), Diarrhea (1 week), Conditions affecting stool sample provision
Sun et al., 2020 [3]	Field center, Gender, Race, Age, Education, Physical activity, Smoking habits, Diet quality, Antihypertensive medication use, BMI, Waist circumference	Pregnancy, Recent Antibiotic use (1 month), Inflammatory bowel disease, Gastrointestinal illness (1 week)

Supplementary Table S1. Adjustments and Exclusion criteria of the included studies.

Nakai et al., 2021 [5]	Age, BMI, Dietary fiber intake	Gastrointestinal disease, Diabetes, Chronic kidney disease, Recent Antibiotic/Probiotic use (3 months)
Wan et al., 2021 [27]	Gender, Age, BMI	Recent Antibiotic/Probiotic use (30 days), Digestive system disease, Gastrointestinal surgery, Cardiovascular diseases, Stroke, Kidney failure, Cancer, Diabetes
Wang JM et al., 2021 [28]	Age, Gender, BMI, Pulse, FBG, TC, RBC count, WBC count	Secondary hypertension, Cardiovascular, Digestive, Respiratory diseases, Cancer, Epilepsy, Sleep Apnea, Psychiatric symptoms, Pregnancy, Laboratory abnormalities, Recent drug use, Participation in trials
Liu Y et al., 2021 [29]	Gender, BMI, Triglycerides, TC, HDL, LDL, Smoking habits, Alcohol use	Recent Antibiotic use (3 months), Inflammatory bowel disease, Tumors of digestive system, Gastrointestinal surgery, Recurrent diarrhea or constipation (1 month)
Wang Y et al., 2021 [17]	Age, Gender, Provinces, Urbanization index, Income, Education level, Energy intake, Animal-source food, Sodium intake, Physical activity, Smoking habits, Alcohol use, eGFR, BMI (in some models)	Not specified
Qu et al., 2022 [16]	Age, gender, BMI, Education level	Neurological/psychiatric disorders, Digestive system diseases, Diabetes, Respiratory diseases, Stroke, Recent Antibiotic/Probiotic use, Substance dependence

Abbreviations: HDL, High-Density Lipoprotein; LDL, Low-Density Lipoprotein; RBC, Red Blood Cell; WBC, White Blood Cell; BMI, Body Mass Index; eGFR, estimated Glomerular Filtration Rate; FBG, Fasting Blood Glucose; TC, Total Cholesterol; NSAID, Non-Steroidal Anti-Inflammatory Drug
