# Supplementary material for: The Microbial Perspective: A Systematic Literature Review on Hypertension and Gut Microbiota
Source: Nutrients. 2024 Oct 30;16(21):3698. doi: 10.3390/nu16213698 (PMC11547301; doi:10.3390/nu16213698)
Supplement: Supplementary file 1 [file nutrients-16-03698-s001.zip › Supplementary Table S2.pdf]

Supplementary Table S2. Blood pressure measurement methods and guidelines followed by the included studies.

Study	Blood Pressure Measurement Method	Guidelines
Li et al. 2017 [4]	Blood pressure was measured in triplicate in a seated position, and the average was calculated	American Society of Hypertension & International Society of Hypertension Guidelines
Palmu et al. 2020 [25]	Two measurements were taken in a seated position, and the mean of the two readings was calculated	Not explicitly stated
Verhaar et al. 2020 [26]	Two measurements were taken after 5 minutes of rest in the supine position, with the average calculated	ESC/ESH Guidelines (2018)
Sun et al. 2020 [3]	Blood pressure was measured using an oscillometric device, and the average of the second and third readings was used	Not explicitly stated
Nakai et al. 2021 [5]	Blood pressure was assessed through both office BP measurements and 24-hour ambulatory blood pressure monitoring (ABPM), with the average of multiple readings calculated	ESH Guidelines
Wan et al. 2021 [27]	Blood pressure measurement method not specified	Not explicitly stated
Wang JM et al. 2021 [28]	Blood pressure was assessed using 24-hour ambulatory blood pressure monitoring (ABPM), and the average value was utilized	JNC 7 Guidelines
Liu Y et al. 2021 [29]	Blood pressure was measured in triplicate in a seated position, and the average of the readings was used	Not explicitly stated
Wang Y et al. 2021 [17]	Blood pressure was measured using a mercury sphygmomanometer, with three readings taken, and the average was calculated	ISH Guidelines (2020)
Qu et al. 2022 [16]	Blood pressure measurement method not specified	Chinese Guidelines (2018 Revised)
Yan et al. 2017 [18]	Blood pressure measurement method not provided	Not explicitly stated
Jackson et al. 2018 [7]	Blood pressure measurement method not provided	Not explicitly stated
Dan et al. 2019 [20]	Blood pressure measurement method not provided	Chinese Guidelines (2010)
Takagi et al. 2020 [22]	Blood pressure measurement method not provided	Not explicitly stated
Mushtaq et al. 2019 [19]	Blood pressure was measured using a mercury sphygmomanometer, with three readings taken, and the average was calculated	WHO Blood Pressure Classification
Silveira-Nunes et al. 2020 [24]	Blood pressure was measured at the time of diagnosis, with patients treated for hypertension for more than 10 years included	Brazilian Society Guidelines (2010)<br>-<br>Sociedade Brasileira de Cardiologia/Sociedade Brasileira de Hipertensão/Sociedade Brasileira de Nefrologia (2010).
Calderón-Pérez et al. 2020 [21]	Blood pressure was measured using an automatic sphygmomanometer, with two readings taken, and the average was calculated	ESC/ESH Guidelines (2018)
Zhu et al. 2020 [23]	Blood pressure measurement method not provided	WHO Blood Pressure Classification

Abbreviations: ESC, European Society of Cardiology; ESH, European Society of Hypertension; ISH, International Society of Hypertension; JNC, Joint National Committee; WHO, World Health Organization
