# Supplementary material for: The Microbial Perspective: A Systematic Literature Review on Hypertension and Gut Microbiota
Source: Nutrients. 2024 Oct 30;16(21):3698. doi: 10.3390/nu16213698 (PMC11547301; doi:10.3390/nu16213698)
Supplement: Supplementary file 1 [file nutrients-16-03698-s001.zip › Supplementary Table S3.pdf]

Supplementary Table S3 (A). Critical appraisal of case-control studies (n = 10)

CASP Questions	Yan et al. 2017 [18]	Mushtaq et al. 2019 [19]	Dan et al. 2019 [20]	Takagi et al. 2020 [22]	Zhu et al. 2020 [23]	Silveira-Nunes et al. 2020 [24]	Nakai et al. 2021 [5]	Wan et al. 2021 [27]	Wang JM et al. 2021 [28]	Liu Y et al. 2021 [29]
1. Did the study address a clearly focused issue?	2	2	2	2	2	2	2	2	2	2
2. Did the authors use an appropriate method to answer their question?	2	2	2	2	2	2	2	2	2	2
3. Were the cases recruited in an acceptable way?	1	1	1	1	1	1	1	2	1	1
4. Were the controls selected in an acceptable way?	1	1	1	1	1	1	1	2	1	1
5. Was the exposure accurately measured to minimize bias?	2	1	1	1	1	1	1	1	1	1
6. (a) Aside from the experimental intervention, were the groups treated equally?	2	2	1	1	1	1	2	2	1	2
6. (b) Have the authors taken account of the potential confounding factors in the design and/or in their analysis?	1	1	1	1	1	1	2	1	1	2
7. How large was the treatment effect?	1	1	1	1	1	1	1	1	1	1
8. How precise was the estimate of the treatment effect?	1	1	1	1	1	1	1	1	1	1
9. Do you believe the results?	2	2	2	2	2	2	1	2	1	1
10. Can the results be applied to your patients/the population of interest?	0	0	0	0	0	0	0	0	0	0
11. Do the results of this study fit with other available evidence?	2	2	2	2	2	2	2	2	2	2
Total score	17	16	15	15	15	15	16	18	14	16

Supplementary Table S3 (B). Critical appraisal of cohort studies (n = 7)

CASP Questions	Li et al. 2017 [4]	Jackson et al. 2018 [7]	Palmu et al. 2020 [25]	Verhaar et al. 2020 [26]	Sun et al. 2020 [3]	Wang Y et al. 2021 [17]	Qu et al. 2022 [16]
1. Did the study address a clearly focused issue?	2	2	2	2	2	2	2
2. Was the cohort recruited in an acceptable way?	2	2	2	2	2	2	2
3. Was the exposure accurately measured to minimize bias?	1	2	2	2	2	2	1
4. Was the outcome accurately measured to minimize bias	1	2	2	2	2	2	1
5. (a) Have the authors identified all important confounding factors?	1	1	2	1	2	2	1
5. (b) Have they taken account of the confounding factors in the design and/or analysis?	1	1	2	1	2	2	1
6. (a) Was the follow up of subjects complete enough?	1	1	1	1	1	1	1
6. (b) Was the follow up of subjects long enough?	1	1	1	1	1	1	1
7. What are the results of this study?	2	2	2	2	2	2	2
8. How precise are the results?	2	2	2	2	2	2	2
9. Do you believe the results?	2	2	2	2	2	2	2
10. Can the results be applied to the local population?	0	1	1	1	1	0	0
11. Do the results of this study fit with other available evidence?	2	2	2	2	2	2	2
12. What are the implications of this study for practice?	1	1	1	1	1	1	1
Total score	19	22	24	22	24	23	19

Supplementary Table S3 (C). Critical appraisal of cross-sectional study (n = 1)

CASP Questions	Calderón-Pérez et al. 2020 [21]
1. Did the study address a clearly focused issue?	2
2. Did the authors use an appropriate method to answer their question?	2
3. Were the subjects recruited in an acceptable way?	1
4. Were the measures accurately measured to reduce bias?	1
5. Were the data collected in a way that addressed the research issue?	1
6. Did the study have enough participants to minimize the play of chance?	0
7. How are the results presented and what is the main result?	2
8. Was the data analysis sufficiently rigorous?	2
9. Is there a clear statement of findings?	2
10. Can the results be applied to the local population?	0
11. How valuable is the research?	1
Total score	14

0 = No

1 = Can't tell

2 = Yes
